# Notch Signaling Pathway Was Involved in Regulating Programmed Cell Death 1 Expression during Sepsis-Induced Immunosuppression

**DOI:** 10.1155/2015/539841

**Published:** 2015-04-30

**Authors:** Tingting Pan, Zhaojun Liu, Jianyong Yin, Tianyun Zhou, Jialin Liu, Hongping Qu

**Affiliations:** ^1^Department of Critical Care Medicine, Ruijin Hospital, Shanghai Jiaotong University School of Medicine, 197 Rui-Jin Er Road, Shanghai 200025, China; ^2^Department of Pulmonary Medicine, Ruijin Hospital, Shanghai Jiaotong University School of Medicine, 197 Rui-Jin Er Road, Shanghai 200025, China

## Abstract

Programmed cell death 1 (PD-1) plays an important pathologic role in sepsis-induced immunosuppression. However, whether PD-1 overexpression occurs early during septic shock is unknown and its regulation mechanism is also unknown. Our study investigated the expressions of PD-1/programmed death-ligand 1 (PD-L1) on immune cells in peripheral blood from the early-stage septic shock patients. We found that both PD-1 and PD-L1 showed increased expressions on the CD4^+^ T cells and monocytes. It indicated that PD-1 expression might be an early biomarker to assess illness severity and predict the prognosis of septic shock. Then, we further investigated the mechanism underlying the regulation of PD-1 expression. Our data showed that Notch signaling pathway was activated in both septic shock patients and lipopolysaccharide- (LPS-) tolerant THP1 cells and both interleukin-10 (IL-10) and PD-1 were increased in the THP1 cells. Inhibition of Notch signaling by N-[N-(3,5-difluorophenacetyl)-L-alanyl]-S-phenyl glycinet-butyl ester (DAPT) induced significantly decreased expressions of PD-1 and IL-10 in the LPS-tolerant cell model. Our work suggested that Notch signaling pathway was involved in the regulation of PD-1 expression.

## 1. Introduction

Sepsis, a systemic inflammatory response to infection, is one of the most challenging clinical problems worldwide and the leading cause of death in noncoronary ICUs [1]. Recent studies have shown that both pro- and anti-inflammatory mechanisms may occur early and simultaneously in sepsis [2]. During the proinflammatory phase, septic patients also enter a stage of protracted immunosuppression. Patients in the immunosuppressive phase of sepsis are often unable to control the primary infection and may also acquire a secondary infection during hospitalization. This phenomenon has recently been identified as an important cause of mortality during sepsis [3]. Our previous study showed that the proportion of CD4^+^ CD25^+^Foxp3^+^T cells (Tregs) was significantly elevated in the peripheral blood from early-stage septic shock patients [4]. Tregs are involved in the regulation of peripheral tolerance which demonstrated again that immunosuppression was a key factor in the development of sepsis [5, 6]. All these suggested that understanding the mechanism underlying the immunosuppression will help to identify the novel targets in the treatment of sepsis.

Recently, more and more lines of evidence have revealed the important role of programmed cell death 1 (PD-1) and programmed death-ligand 1 (PD-L1) in the pathogenesis of sepsis [7]. The PD-1, a surface receptor, belongs to the CD28 family and is mainly expressed on activated T cells, natural killer T cells, and myeloid cells [8]. PD-1 interacts with two B7 family ligands, PD-L1 and PD-L2. PD-L1 is widely expressed on antigen presenting cells (APCs) and hematopoietic cells. The interaction between PD-1 and PD-L1 activates an inhibitory pathway that leads to abrogation of cellular response and causes CD8^+^ T cell exhaustion during chronic viral infection [9, 10]. Zhang and colleagues [11] found that in vitro blockade of the PD-1/PD-L1 pathway decreased apoptosis and improved immune cell function in septic patients. There are also three independent groups which showed that blockade of the PD-1 pathway improved survival in clinically relevant animal models of bacterial and fungal sepsis [7, 11, 12]. These studies strongly suggest that PD-1 pathway may become a potential immune-modulatory therapy for sepsis-induced immunosuppression. Therefore, a better understanding of the molecular events controlling PD-1 expression is essential for sepsis. Yet very little is known on the regulation of PD-1 expression during sepsis-induced immunosuppression.

In this study, first we investigated the expressions of PD-1/PD-L1 on both CD4^+^ T cells and monocytes from peripheral blood in the early-stage septic shock patients and showed the relationship between PD-1/PD-L1 and clinical outcomes. Then, we evaluated the Notch signaling pathway, which contributes to the regulation of the peripheral immune cells tolerance through its ability to influence cell survival and growth [13], in the peripheral blood mononuclear cells (PBMCs) from the septic shock patients. Finally, we explored the regulation of PD-1 expression by inhibiting the Notch signaling pathway in a LPS-tolerant THP1 cells model. Here, for the first time, we demonstrated that Notch signaling pathway was activated in the early-stage septic shock patients and involved in the regulation of PD-1 expression.

## 2. Methods

### 2.1. Patients and Ethics Statement

The patients enrolled in this observational/clinical study were recruited from the surgical, emergency, and respiratory ICUs of Ruijin Hospital, Shanghai Jiaotong University (Shanghai, China). 30 healthy individuals matched with sex and age were enrolled into control group. The study protocol was approved by the Ruijin Hospital Ethics Committee, Shanghai Jiaotong University School of Medicine, China. The individuals in this paper have given written informed consent to publish these case details. Sepsis was diagnosed according to criteria established by the American College of Chest Physicians/Society of Critical Care Medicine [14]. Vasopressive therapy was initiated at the time of diagnosis of septic shock. The following information was collected: demographic characteristics (age and gender), APACHE II score (Acute Physiology and Chronic Health Evaluation II) at inclusion, white blood cell count at inclusion, site of infection, microbiological findings, and the outcome after 28 days (nonsurvival or survival). Study exclusion criteria included patients aged < 18 years; more than 24 hours from sepsis diagnosis to blood collection; preexisting cancer; an absence of circulating leukocytes; the presence of preexisting hematological or immunological disease; patients with HIV or HBV.

### 2.2. Blood Collection and Processing

Peripheral vein blood samples were obtained from each patient, diagnosed with septic shock within 24 hours, and the healthy individuals. The blood samples were transported to the clinical research center at 4°C within 1 hour. To detect the expressions of PD-1 and PD-L1 on the immune cells by flow cytometry, peripheral blood mononuclear cells (PBMCs) were isolated by Ficoll density gradient centrifugation.

### 2.3. Flow Cytometry

Blood samples were obtained from 56 patients with septic shock and 30 healthy volunteers. After lysing red cells with FACS-lysing solution (BD Biosciences; San Jose, CA, USA), the immune cells were stained with FITC-labeled anti-CD4 (Clone A161A1) or FITC-labeled anti-CD14 (Clone HCD14) and PE-labeled anti-PD-1 (Clone EH12.2H7) or PE-labeled anti-PD-L1 antibodies (Clone 29E.2A3) (Biolegend; San Diego, CA, USA). The stained cells were analyzed by using a FACS Calibur flow cytometer (Becton-Dickinson, BD Biosciences) and CELLQUEST software.

### 2.4. Cell Culture and Endotoxin Tolerance Induction

THP-1 cells were purchased from the Type Culture Collection of the Chinese Academy of Sciences, Shanghai, China [15]. Cells were maintained under conditions of 37°C and 5% CO_2_ in RPMI-1640 media supplemented with penicillin G (10 U/mL), streptomycin (10 *μ*g/mL), L-glutamine (2 mM), and 10% fetal bovine serum (FBS) (Hyclone Laboratories, Logan, UT, USA). THP1 cells were pretreated with 1.0 *μ*g/mL Gram-negative LPS (*Escherichia coli* 0111:B4; Sigma-Aldrich, St. Louis, MO, USA) for 16 h. Then, nontreated and pretreated THP-1 cells were pelleted, washed once in PBS, and resuspended in culture media at 5.0–10.0 × 10^5^ cells/mL. Finally, cells of both groups were stimulated with LPS (1.0 *μ*g/mL) for another 6 hours to make LPS-nontolerant and LPS-tolerant models [16]. The control group was not given any stimulation. After above treatment, cells were washed for three times and total RNA was extracted. Reduced proinflammatory cytokines (mainly TNF-*α*) and increased immunosuppression mediators (mainly IL-10) are always used to evaluate whether the endotoxin tolerant model is set up successfully. In our study, the TNF-*α* and IL-10 expressions were examined by using real-time PCR to verify the in vitro model of endotoxin tolerance.

Additionally, in LPS-tolerant group, gamma secretase inhibitor N-[N-(3,5-difluorophenacetyl)-L-alanyl]-S-phenyl glycinet-butyl ester (DAPT) (Sigma, St. Louis, MO, USA, 40 *μ*M) was added into the culture at the beginning of the treatment of LPS to inhibit the Notch signaling pathway. Low passage number and log-phase cells were used for all experiments.

### 2.5. Total RNA Extraction, cDNA Synthesis, and Real-Time PCR

Total RNA was extracted from patient PBMCs and THP1 cells by using TRIzol reagent (Invitrogen; Grand Island, NT, USA) according to the manufacturer's instructions. RNA (2 *μ*g) was reverse transcribed by using reverse transcriptase with random hexamers as primers (PrimeScript TM RT-PCR Kit, Takara, Kyoto, Japan). The resulting complementary DNAs (cDNAs) were diluted 2-fold with DNase/RNase-free water. Real-time quantitative PCR was performed by using a 7500 Fast Real-Time PCR system (Applied Biosystems; Foster City, CA, USA) and SYBR Premix Ex Taq (Takara, Kyoto, Japan) under the following conditions: denaturation at 95°C for 30 sec, followed by 40 cycles at 95°C for 5 sec, 60°C for 34 sec, and then a dissociation phase. *β*-actin was used as an endogenous control to normalize for RNA levels and efficiency of the reverse-transcription reaction. The primer sequences used are described in Table 1.

### 2.6. Data Analysis and Statistics

The clinical and biological parameters of patients are presented as frequencies, percentages, medians, or interquartile ranges (IQRs). Experiment data are presented as the median value with interquartile ranges or as the mean ± SEM. Normally distributed data were analyzed by using two-tailed Student's *t*-test, and nonnormally distributed data were analyzed by using the Mann-Whitney *U* test. *P* values < 0.05 were considered statistically significant. All statistical analyses were conducted by using Prism 4.0 software (GraphPad Software, La Jolla, CA, USA).

## 3. Results

### 3.1. Clinical Characteristics of the Patients with Early Stage of Septic Shock

Fifty-six patients at early stage of septic shock (24 females and 32 males) were included in this study. Demographic information for the enrolled patients is shown in Table 2. The median age of the patients was 59 years (range 54 to 63 years), and the median APACHE II score was 16 (range 14 to 23). Patients with septic shock showed increased numbers of white blood cells compared to healthy volunteers. 29 septic shock patients were infected with Gram-negative bacilli, 22 patients were infected with Gram-positive cocci, and 5 patients were infected with fungi. The main infection sites among them were the lungs and abdomen. Twenty-five of the 56 patients died within 28 days after diagnosis of septic shock.

### 3.2. PD-1 and PD-L1 Expressions Were Increased in Patients with Early Stage of Septic Shock

**Figure 1:**
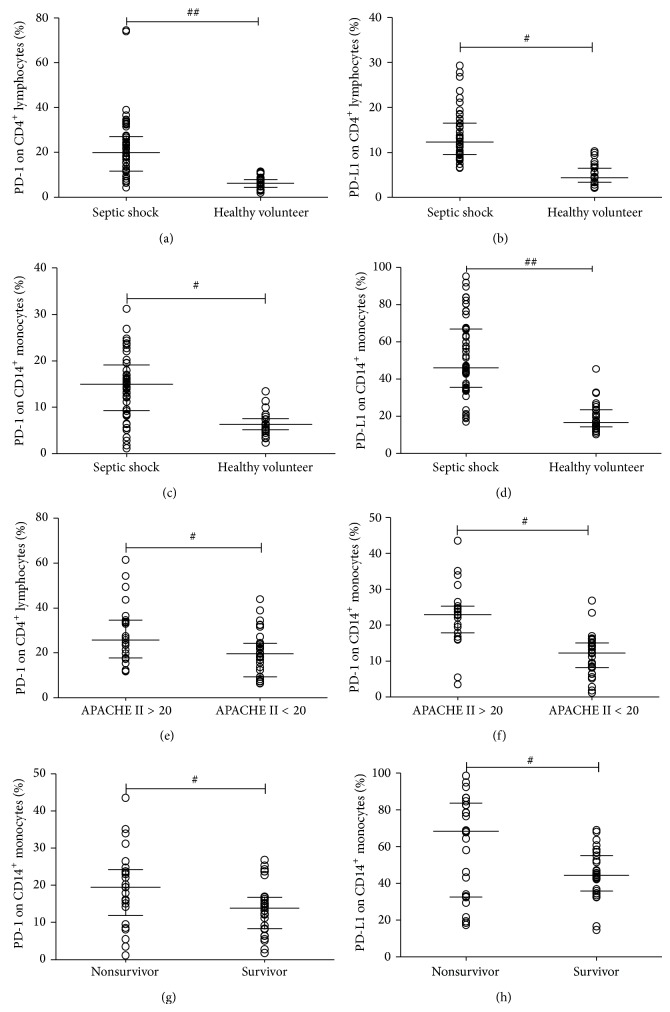
Expressions of PD-1/PD-L1 on CD4^+^ T cells and monocytes in early-stage septic shock patients. Percentages of PD-1 and PD-L1 expressions on CD4^+^ T cells and monocytes were compared between septic shock patients (*n* = 56) and healthy volunteers (*n* = 30): (a) percentage of PD-1 expression on CD4^+^ T cells; (b) percentage of PD-L1 expression on CD4^+^ T cells; (c) percentage of PD-1 expression on monocytes; (d) percentage of PD-L1 expression on monocytes. The percentages of PD-1 expression on CD4^+^ T cells (e) and monocytes (f) were compared between patients with APACHE II scores > 20 (*n* = 22) and < 20 (*n* = 34). These expressions on monocytes were also compared between survivors (*n* = 31) and nonsurvivors (*n* = 25): (g) percentages of PD-1 expression; (h) percentages of PD-L1 expression. Each dot represents one individual value. Analyses were conducted using the Mann-Whitney rank sum test. ^#^ *P* < 0.05, ^##^ *P* < 0.01.

Expressions of both PD-1 and PD-L1 on CD4^+^ T cells and monocytes were measured in each patient diagnosed with septic shock within 24 hours (Figure 1). CD4^+^ T cells obtained from patients showed significantly greater expressions of both PD-1 and PD-L1 compared to CD4^+^ T cells obtained from healthy volunteers (21.59% versus 6.37%, *P* < 0.001, and 13.67% versus 4.97%, *P* < 0.01, resp.) (Figures 1(a) and 1(b)). Additionally, PD-1 and PD-L1 expressions on monocytes (Figures 1(c) and 1(d)) were also much higher in patients compared to healthy volunteers (for PD-1: 14.91% versus 6.89%, *P* < 0.01; for PD-L1: 51.82% versus 17.12%, *P* < 0.01).

### 3.3. PD-1 Expression Might Be an Early Biomarker to Assess Illness Severity and Predict the Prognosis of Septic Shock

Here, we assessed the association between PD-1/PD-L1 expressions and illness severity and found that patients with an APACHE II score > 20 showed significantly greater PD-1 expression on CD4^+^ T cells and monocytes compared to patients with an APACHE II score < 20 (for CD4^+^ T cells: 28.68% versus 19.67%, *P* < 0.01, Figure 1(e); for monocytes: 22.55% versus 11.73%, *P* < 0.01, Figure 1(f)). However, there were no differences in PD-L1 expression on CD4^+^ T cells and monocytes obtained from patients with different levels of physical dysfunction (data not shown). Then, we analyzed the association between PD-1/PD-L1 expression and patient prognosis by comparing expression levels of PD-1 and PD-LI on CD4^+^ T cells and monocytes obtained from sepsis survivors and nonsurvivors. Nonsurvivors showed higher levels of both PD-1 and PD-L1 expressions on monocytes (for PD-1: 19.13% versus 13.79%, *P* < 0.05, Figure 1(g); for PD-L1: 60.21% versus 45.06%, *P* < 0.01, Figure 1(h)). However, survivors and nonsurvivors showed similar levels of PD-1 and PD-L1 expressions on CD4^+^ T cells (data not shown).

### 3.4. Expressions of PD-1 and Notch Signaling Pathway Were Increased in LPS-Tolerant THP1 Cells

**Figure 2:**
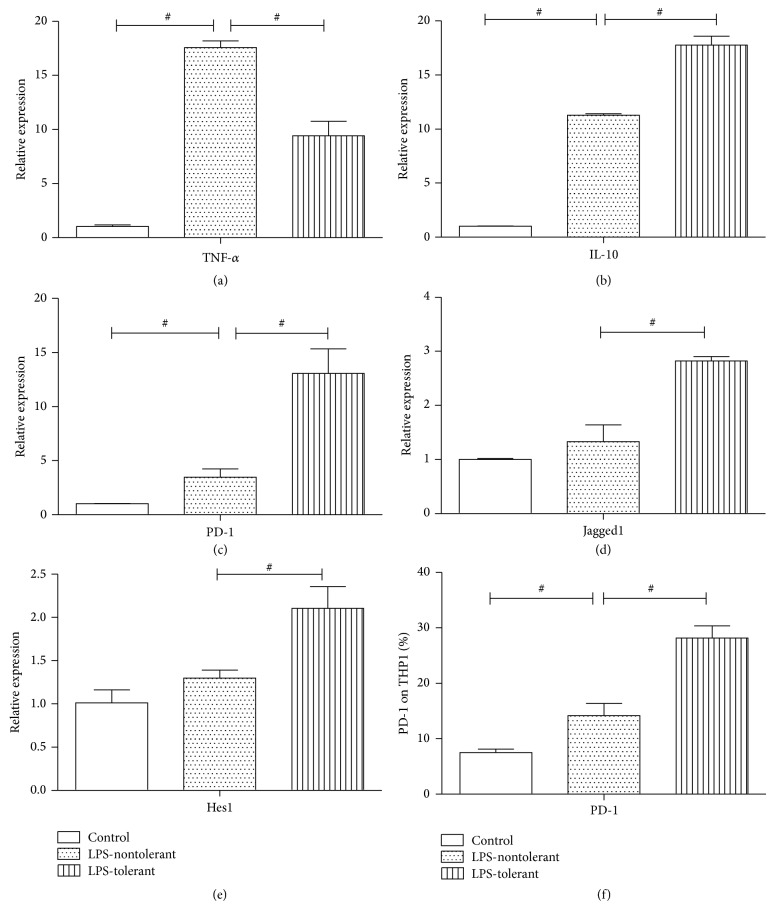
PD-1 and Notch-related molecules levels in LPS-tolerant THP1 cells. THP1 cells were pretreated with 1.0 *μ*g/mL LPS for 16 h. Then, nontreated and pretreated THP-1 cells were pelleted, washed once in PBS, and resuspended in culture media at 5.0–10.0 × 10^5^ cells/mL. Finally, cells of both groups were stimulated with LPS (1.0 *μ*g/mL) for another 6 hours to make LPS-nontolerant and LPS-tolerant models. The control group was not given any stimulation. After above treatment, cells were washed for three times and total RNA was extracted. The mRNA levels of TNF-*α* (a), IL10 (b), PD-1 (c), Jagged1 (d), and Hes1 (e) and protein levels of PD-1 (f) were examined in control, LPS-tolerant, and LPS-nontolerant groups. Data shown are normalized to *β*-actin mRNA and presented as 2^−ΔΔCT^. ^#^ *P* value < 0.05.

Notch signaling is a key pathway to control cell-fate choice in a large number of cell types including immune cells. The role of the Notch signaling pathway in peripheral tolerance leads us to evaluate its expression in endotoxin tolerance, which is a hallmark of sepsis-induced immunosuppression. The decreased mRNA level of proinflammatory cytokine TNF-*α* and increased mRNA level of anti-inflammatory cytokine IL-10 were proved in a LPS-tolerant THP1 cell model (Figures 2(a) and 2(b)). The PD-1 mRNA levels were markedly increased in LPS-tolerant cells compared to those in LPS-nontolerant cells (*P* < 0.05, Figure 2(c)). We also measured the mRNA levels of Notch signaling proteins including Jagged1 and Hes1 and found that, compared to LPS-nontolerant cells, Jagged1 and Hes1 expressions were much higher in LPS-tolerant cells (*P* < 0.05, Figures 2(d) and 2(e)). These data suggest that mRNA levels of PD-1 and Notch signals were increased and there may be an association between the Notch signaling pathway and PD-1 expression in the endotoxin tolerance. In addition, the results of PD-1 were proved in protein levels (*P* < 0.05, Figure 2(f)).

### 3.5. Notch Signaling Pathway Was Activated in Patients at Early Stage of Septic Shock

**Figure 3:**
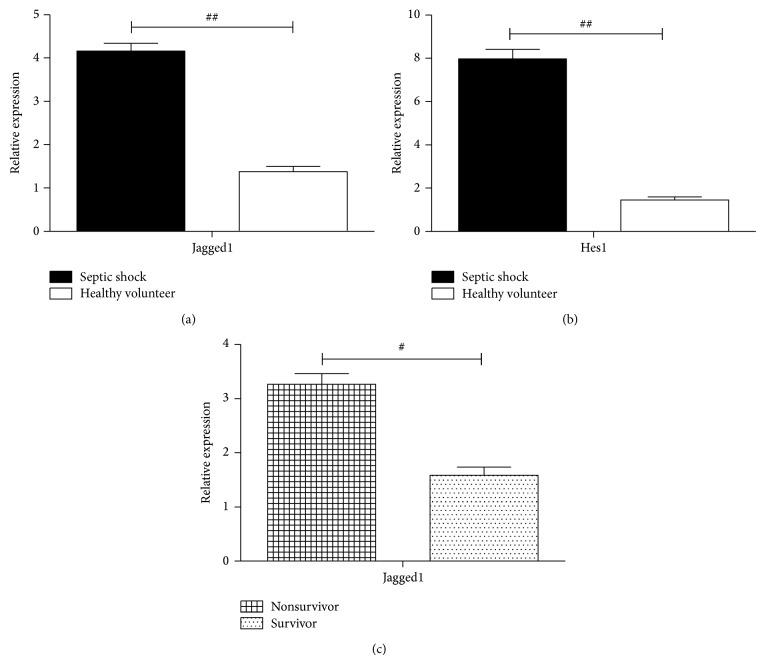
Notch molecules mRNA levels in PBMCs from patients at early stage of septic shock. Total RNA was extracted from PBMCs of patients within 24 hours once septic shock was diagnosed. The mRNA levels of Jagged1 (a) and Hes1 (b) were compared between patients and healthy volunteers. (c) Jagged1 mRNA level was compared between survivors and nonsurvivors with septic shock. Data are normalized to *β*-actin mRNA and presented as 2^−ΔΔCT^. ^#^ *P* < 0.05, ^##^ *P* < 0.01.

To approve the activation of the Notch signaling pathway during sepsis, we measured the mRNA levels of Notch signal proteins in PBMCs obtained from patients at early stage of septic shock. We found that mRNA levels of Jagged1 and Hes1 in patients were much higher compared to those in healthy volunteers (*P* < 0.05) (Figures 3(a) and 3(b), resp.). Additionally, among the septic shock patients, mRNA levels of Jagged1 in nonsurvivors were higher than those in survivors (Figure 3(c)).

### 3.6. Notch Signaling Pathway Regulates PD-1 Expression in LPS-Tolerant THP1 Cells

**Figure 4:**
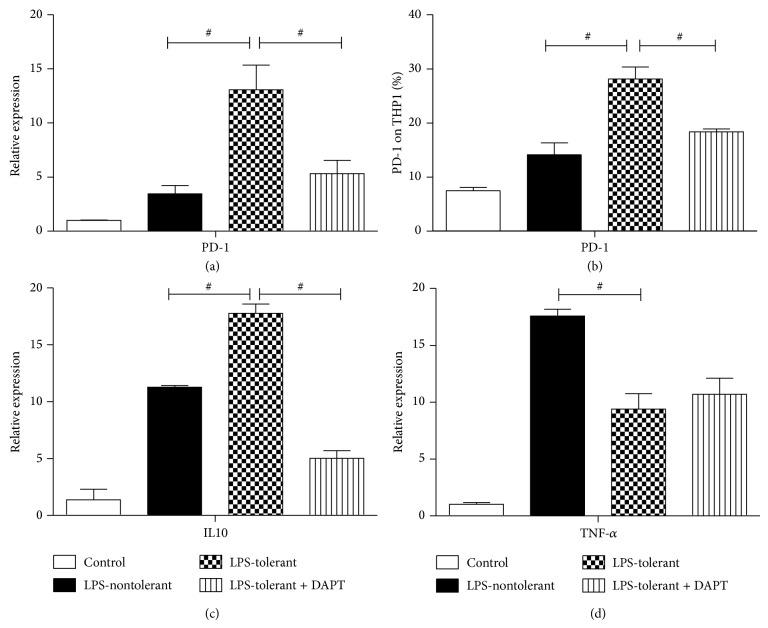
Notch inhibition by DAPT decreased PD-1 and IL-10 transcriptions in LPS-tolerant THP1 cells. LPS-tolerant THP1 cells were induced as mentioned above. In tolerant group, DAPT (40 *μ*M) was added into the culture at the beginning of the treatment of LPS to inhibit the Notch signaling pathway. The mRNA levels of PD-1 (a), IL-10 (c), and TNF-*α* (d) and protein levels of PD-1 (b) were examined in control, LPS-nontolerant, LPS-tolerant, and DAPT groups. Data are normalized to *β*-actin mRNA and presented as 2^−ΔΔCT^. ^#^ *P* value < 0.05.

To explore the role of the Notch signaling pathway in PD-1 expression, a gamma secretase inhibitor DAPT was used to block Notch signaling pathway. After inhibition of the Notch signaling by DAPT, both mRNA and protein levels of PD-1 were significantly decreased in the LPS-tolerant cells (both *P* values < 0.05, Figures 4(a) and 4(b)). And mRNA levels of IL10 were also decreased when Notch signaling pathway was blocked in the LPS-tolerant cells (*P* values < 0.05, Figure 4(c)). TNF-*α* mRNA levels were increased after blockading the Notch signaling pathway (*P* values > 0.05, Figure 4(d)).

## 4. Discussion

Previous studies suggested that both proinflammatory and anti-inflammatory processes begin promptly after sepsis initiation. It has been reported that PD-1 and its ligand PD-L1 deliver inhibitory signals capable of inducing immune tolerance [8] and prolonged overexpression of PD-1 might result in T cell dysfunction in sepsis patients [17, 18]. Here, we have shown overexpressions of PD-1 and PD-L1 on monocytes and CD4^+^ lymphocytes obtained from patients at early stage of septic shock. It not only demonstrated the role of PD-1 and PD-L1 in the pathogenesis of sepsis but also suggested that the early upregulated PD-1 might be a useful marker to evaluate the immune state of sepsis patients. Furthermore, the present work showed that PD-1 expression on immune cells was much higher in nonsurvival septic shock patients than survivors. Recent studies suggested that, in a murine model of sepsis, administration of PD-1 antibody markedly diminished sepsis-induced apoptosis and improved survival and it also showed an enhanced bacterial clearance capacity after induction of sepsis by cecal ligation and puncture (CLP) in PD-1^−/−^ mice [7, 19]. All above lines of evidence suggested that PD-1 could assess illness severity, predict the prognosis of sepsis, and be a potential target for the treatment of sepsis-induced immunosuppression. Therefore, it is important to find out the regulation mechanism for this novel approach during sepsis.

Recently, Notch signaling pathway was identified to induce PD-1 expression on T cells maintaining them in an exhausted state during chronic viral infection [20]. In mammals, four Notch receptors (Notch 1–Notch 4) and five ligands (Jagged1, Jagged2, Delta-like-1, Delta-like-3, and Delta-like-4) have been identified [21]. Notch downstream target genes include members of the basic helix-loop-helix family (Hes1 and Hes5) and the hairy and enhancer of split-related (HESR) family (Hey1 and Hey2) [21, 22]. During the peripheral tolerance, Jagged1 and Hes1 have been reported to be involved in inhibiting primary and secondary immune responses [23, 24]. In our experiment, the decreased TNF-*α* and increased IL-10 transcriptomic responses to two subsequent LPS challenges demonstrated that endotoxin tolerance THP1 cell model was established successfully. We found that the expressions of Jagged1 and Hes1 in LPS-tolerant cells were significantly higher than LPS-nontolerant cells. This reproduced ex vivo observation was also found in the septic shock patients. Here, we demonstrated that Notch signaling pathway was activated in both early septic shock patients and THP1 cells response to two subsequent LPS challenges. The specific Notch ligands can be induced by pathogen-derived signals on the differentiation or function of CD4^+^ T cells and haematopoietic cells. Previous study suggested that the Notch signaling Jagged1 was induced by LPS occurring in a JNK-dependent manner [25]. Therefore, the septic shock patients infected with bacteria, especially G-bacteria, may activate the Notch signaling pathway via JNK-dependent manner induced by LPS.

It has been reported that, during chronic virus infection, the inhibition of PD-1 expression by the highly specific inhibitor SAHM1 showed that Notch signaling directly controls PD-1 via P*dcd*1 transcription in activated CD8^+^ T cells [20]. Here, the inhibition of Notch signaling pathway induced significantly decreased PD-1 expression in the LPS-tolerant cell model. We hypothesized that the activated Notch signaling pathway may be involved in the expression of PD-1 during sepsis. The mechanism leading to the regulation of PD-1 by Notch signaling pathway in LPS-tolerant THP1 cells may be the similar directly regulated by varied transcription factors involved in THP1 monocytes.

In current experiment, we also found that both PD-1 and IL-10 were increased in LPS-tolerant cells. After inhibition of Notch pathway by DAPT, IL-10 and PD-1 were decreased simultaneously. Recent studies noted the interaction between them: PD-1 could enhance IL-10 production [26] and IL-10 also induced PD-1 expression in monocytes and CD4^+^ T cells [27]. Additionally, previous research showed that DC matured by Jagged1 (Jgd1-conditioned DC) produced the high level of IL-10 through TLR (via LPS) signaling [28]. However, whether the Notch signaling pathway in regulation of PD-1 expression is through the IL-10 during sepsis-induced immunosuppression is unclear.

Our study has some limitations which should be mentioned. First, our results need to be confirmed in a larger multicenter study. Second, our findings concerning the role of Notch signaling in PD-1 and IL-10 expressions under conditions of LPS tolerance are mainly at the gene transcription level and should be further confirmed at the protein level. Third, our current study was very preliminary and exploratory in nature, and the mechanisms responsible for Notch regulation of PD-1 expression on PBMC under conditions of endotoxin tolerance require further investigation in a dedicated study.

In summary, our data showed that the enhanced PD-1 could be an early biomarker to assess not only the proper immune phase but also the illness severity and prognosis for the septic shock patients. Noticeably, we also found that Notch signaling pathway was activated during the early stage of the septic shock and involved in regulation of PD-1 expression. Thus it revealed a possible regulation mechanism of PD-1, which may be a promising target for the therapeutic intervention in sepsis and in the field of infectious disease. It may be helpful to improve the potential immunomodulatory strategies.

## Figures and Tables

**Table 1:** Primer sequences used in the real-time quantitative polymerase chain reaction.

Gene	Forward primer	Reverse primer
*β*-actin	AAG GTG ACA GCA GTC GGT T	TGT GTG GAC TTG GGA GAG G
PD-1	GGA AAC CCC TCC ACC TTT A	TCT GCC TGC CCG CTT ACT 3
PD-L1	GGC TGA GCA AGG CAC ATA G	CAC CAC AAG GAG GAG TTA G
*Hes1 *	CCT ATT ATG GAG AAA AGA C	GAG GTG CTT CAC TGT CAT T
*Jagged1 *	CTG GGC TTT GAG TGT GAG T	CCG TGG GAA CAG TTA TTA G
TNF-*α*	CCC AGG GAC CTC TCT CTA ATCA	GCT ACA GGC TTG TCA CTC GG
IL-10	AAT AAG GTT TCT CAA GGG GCT	AGA ACC AAG ACC CAG ACA TCA A

**Table 2:** Clinical characteristics of the patients with early stage of septic shock.

Parameters	Patients with sepsis (*n* = 56)
Females, number (%)	24 (42.86)
Males, number (percentage)	32 (57.14)
Age, median (IQR), year	59 (54, 63)
APACHE II^a^ at inclusion, median (IQR)	16 (14, 23)
WBC, median (IQR), cells/uL	13.84 (11.72, 16.03)
Infection, number (percentage)	
Microbiologically documented	
Bacilli Gram-negative	29 (51.79)
Cocci Gram-positive	22 (39.3)
Fungi	5 (8.93)
Site of infection	
Lungs	28 (50)
Abdomen	20 (35.71)
Others	8 (14.29)
Mortality, number (percentage)	25 (44.64)

Data presented as number of cases and percentage for classification variables or as median and interquartile range (IQR) for continuous variables.

^a^APACHE: Acute Physiology and Chronic Health Evaluation.
